# The immune phenotypes and different immune escape mechanisms in colorectal cancer

**DOI:** 10.3389/fimmu.2022.968089

**Published:** 2022-08-10

**Authors:** Yihao Mao, Yuqiu Xu, Jiang Chang, Wenju Chang, Yang Lv, Peng Zheng, Zhiyuan Zhang, Zhiqiang Li, Qi Lin, Wentao Tang, Dexiang Zhu, Meiling Ji, Guodong He, Qingyang Feng, Jianmin Xu

**Affiliations:** ^1^ Department of General Surgery, Zhongshan Hospital, Fudan University, Shanghai, China; ^2^ Shanghai Engineering Research Center of Colorectal Cancer Minimally Invasive, Shanghai, China; ^3^ Department of General Surgery, No.2 Hospital, Nanping, China

**Keywords:** immune subtypes, infiltration pattern, immune escape, tumor microenvironment, colorectal cancer

## Abstract

The tumor microenvironment (TME) plays a crucial role in tumor progression and metastasis. However, the immune phenotypes of colorectal cancer (CRC) and the underlying immune escape mechanism have not been studied sufficiently. A total of 1802 and 619 CRC samples from the microarray and TCGA cohorts were enrolled, respectively. The ssGSEA algorithm and unsupervised clustering were used for TME cell infiltration speculation and immune phenotype recognition in the above cohorts. A total of 447 samples from Zhongshan Hospital were collected for validation. Immunohistochemistry was performed in this cohort to quantify TME cell infiltration. The single-cell RNA-seq (scRNA-seq) data of 252,940 cells from 60 CRC samples was analyzed for further mechanistic exploration. CRC samples can be classified into three distinct immune phenotypes. Subtype 1, the immune-active subtype, was characterized by high infiltration of activated adaptive immune cells. Subtype 2, the immune-desert subtype, featured high tumor purity and low infiltration of immune and stromal cells. Subtype 3, the stroma-rich subtype, had high infiltration of stromal cells. The stroma-rich subtype conferred a significantly worse prognosis. The three subtypes had different immune escape mechanisms. The immune-active subtype has the highest immune checkpoint expression level. In comparison, the immune-desert subtype had the lowest immunogenicity and defective antigen presentation. The stroma-rich subtype lacked activated immune cells. In conclusion, distinct immune phenotypes and immune escape mechanisms may provide inspiration and direction for further research on CRC immunotherapy.

## 1 Introduction

Colorectal cancer (CRC) is estimated to be the third most commonly diagnosed and cause of death in both men and women in the USA in 2022 (1). The tumor microenvironment (TME), defined as the surrounding environment of a tumor, comprises tumor cells, immune cells, stromal cells, blood vessels, and other mesenchymal cells. The TME plays a crucial role in tumor progression and metastasis (2).

Researchers have focused on the CRC TME from multiple perspectives. Multiple TME cells were found to have prognostic value and predict therapeutic benefit (3, 4). As a classic example, Immunoscore, which quantifies the *in situ* T cell infiltration in CRC tissue, was validated to be a valuable prognostic factor (5) and was recommended in the European Society for Medical Oncology (ESMO) guidelines (6). However, numerous studies have only focused on one or a few types of TME cells. Moreover, most of them were based on pathology levels with limited sample sizes. Confusing, even contradictory results were obtained. As CRC is a highly heterogeneous cancer, we assumed that the same type of TME cell might play different roles in different CRC tissues.

Recently, emerging papers have explored the CRC TME using bioinformatic-based methods, such as CIBERSORT (7), MCPcounter (8), TIMER (9), etc. However, because of the limitations of the above methods, such as only relative infiltration results provided or limited predictable cell types, the landscape of TME cell infiltration patterns in CRC samples has not been systematically clarified to date, nor has the immune escape mechanisms elucidated.

In this study, we comprehensively explored the TME cell infiltration pattern in four independent cohorts. CRC samples can be divided into three immune phenotypes with distinct TME cell infiltration patterns and their underlying immune escape mechanisms. Additionally, the different clinical, prognostic, and genomic characteristics were described.

## 2 Materials and methods

### 2.1 Study population

Four cohorts were enrolled in this research, as illustrated in **Figure S1**. The microarray cohort was composed of 1802 CRC samples from the GSE14333 (10), GSE17538 (11), GSE33113 (12), GSE37892 (13), GSE38832 (14), GSE39084 (15), GSE39582 (16), and KFSYSCC datasets, which are publicly available. The dataset selection criteria were as follows: (1) transcriptomic data, including microarray data using Affymetrix HG-U133A (GEO accession number GPL96) or HG-U133 Plus 2.0 (GEO accession number GPL570) platforms or RNA-Seq data, were available; (2) basic clinicopathological information, including AJCC/UICC TNM stage and survival information (overall survival (OS) or disease-free survival (DFS)) was available; (3) the sample size was larger than 50. The transcriptome and clinical data of GSE14333, GSE17538, GSE33113, GSE37892, GSE38832, GSE39084, and GSE39582 were downloaded from the Gene Expression Omnibus (GEO) repository (https://www.ncbi.nlm.nih.gov/gds/). The expression matrix and clinical information of KFSYSCC were acquired from the supplementary material of a previous publication (17). The TCGA cohort included 619 CRC samples from the TCGA-COAD and TCGA-READ datasets (18). The RNA-Seq (RSEM normalized), copy number variation (CNV), mutation, images of pathology slides, and clinical data were downloaded from Genomic Data Commons (GDC, https://portal.gdc.cancer.gov/) in Dec. 2019. The Zhongshan cohort consisted of 447 consecutive CRC patients who received radical primary tumor resections without prior treatment in Zhongshan Hospital (Shanghai, China) from 2008 to 2009. Demographics and clinical data were collected retrospectively. Cancer stages were determined according to the 8th edition of the AJCC/UICC TNM classification. The scRNA-seq cohort was composed of scRNA-seq data of 252940 cells from 60 CRC samples with clinicopathological information and cell annotation available. Such data were retrieved from a previous high-quality publication (19).

The microarray cohort was utilized to discover the infiltration pattern of TME cells. Subsequent pattern validation was conducted in the TCGA and Zhongshan cohorts. Due to the relatively shorter follow-up time of the TCGA cohort (median follow-up time, 21.9 months), survival-related analysis was performed in the microarray and Zhongshan cohorts (median follow-up time, 49.0 and 62.5 months, respectively). Further mechanistic exploration was performed in the TCGA and scRNA-seq cohorts. This study was approved by the Clinical Research Ethics Committee of Zhongshan Hospital, Fudan University. Informed consent was obtained from all patients in the Zhongshan cohort for the acquisition and use of tissue samples and clinical data.

### 2.2 Microenvironmental cell infiltration speculation and validation

We used the voom function [in R package “limma” (20)] to transform the RSEM normalized TCGA RNA-Seq expression matrix. The ComBat algorithm [“ComBat” function in R package “sva” (21)] was applied to calibrate the heterogeneity among different datasets in microarray and TCGA datasets. The PCA plots illustrating the effect of calibration were shown in **Figure S2**. We used the ssGSEA algorithm [“gsva” function in R package “GSVA” (22)] for infiltration speculation of 31 microenvironmental cells based on transcriptomic data as previously described (23, 24). The gene signature used in our study was mainly based on the signature described in CHAROENTONG P et al. (25), XIAO Y et al. (23) (which was a modification of the LM22 signature of CIBERSORT), and MCPcounter (8). The detailed gene signature is described in **Table S1**.

To validate the accuracy of our methods, we compared our calculation results with the estimation of the cell abundances by CIBERSORT and MCPcounter (**Tables S2 and S3**). The comparison results showed high accordance between methods.

### 2.3 Pseudo-bulk analysis

We applied a pseudo-bulk approach to infer microenvironmental cell infiltration in the scRNA-seq cohort. Raw UMI counts for each gene were summed across each sample and resulted in sample-level UMI counts using the AggregateExpression function (in R package “Seurat” (26)) as previously described (27). Genes with less than 50 reads across samples were excluded from further analysis. The sample-level expression matrix was averaged and voom-transformed for further infiltration speculation as described above.

### 2.4 Unsupervised clustering based on the TME cell infiltration pattern

For TME cell infiltration pattern discovery and validation, a nonnegative matrix factorization algorithm (NMF) (“nmf” function in R package “NMF” (28)) was performed for unsupervised clustering on the scaled ssGSEA results. We used the “nmfEstimateRank” function (30 runs) to choose the optimal number of clusters based on the Cophenetic correlation coefficient changing. The highest clustering number before the Cophenetic correlation coefficient dropping most was selected as the optimal clustering number.

### 2.5 Immunohistochemistry and slide image analysis

Immunohistochemistry (IHC) and hematoxylin and eosin (HE) staining were performed on formalin-fixed, paraffin-embedded TMA as described previously (29). The following cells and their corresponding markers were evaluated by IHC: CD4+ T cells (CD4, BX50023, Biolynx), CD8+ T cells (CD8, M7103, Dako), B cells (CD19, #90176, CST), Treg cells (FOXP3, MAB8214, R&D), mast cells (mast cell tryptase, ab2378, Abcam), macrophages (CD68, GA609, Dako), fibroblasts (α-SMA, GM085102, GeneTech), and endothelial cells (VWF, ab6994, Abcam). The infiltration of each cell was recorded as the mean number of cells in tumor tissue from three random high-power fields (HPF, 200×).

HE stained diagnostic slides of the TCGA dataset were retrieved from the GDC Data Portal. The infiltration of tumor-infiltrating lymphocytes (TILs) was recorded as the mean number of lymphocytes in tumor tissue from three random HPFs. Furthermore, the infiltration of stromal cells was evaluated as the mean proportion of stromal cells in tumor tissue from three randomized HPFs. Tumor necrosis in HE slides was characterized by degraded tumor cells, presented as amorphous coagulum with nuclear debris (**Figure 3G**) (30). Pathologists annotated the necrosis area in each diagnostic slide and the proportion of necrosis was calculated by ImageJ (US National Institutes of Health, Bethesda, MD, USA). The above results were all assessed by two independent pathologists who were blinded to the clinical data and the results were averaged.

### 2.6 Single-cell RNA sequencing data analysis

Pre-processed expression transcript count matrices of 371,223 tumor and adjacent normal cells from 62 CRC patients were downloaded from the GEO repository [GSE178341 (19)]. After excluding cells from tumor samples sorted by CD45 MACS and normal samples, 252,940 cells from 60 CRC samples were utilized for further analysis. The main scRNA-seq analysis was performed using Seurat v4.0.2 (26). The data were normalized, scaled and principal components computed. The Uniform Manifold Approximation and Projection (UMAP) method was used for dimensional reduction. Cell clusters were identified mainly by referring to the cluster annotations provided by the authors. The differentially expressed genes of the specific cluster were calculated by the FindMarkers function. Gene set enrichment analysis (GSEA) was performed on the results of the differential gene expression analysis by clusterProfiler 4.0 (31). Gene Set Variation Analysis (GSVA) was conducted between two cell clusters by the “gsva” function (in R package “GSVA” (22)) based on 50 hallmark gene sets in MSigDB 7.5.1 (32).

### 2.7 Genomic analysis in TCGA cohort

TCGA level 3 mutation data were downloaded from the GDC Data Portal. The mutation load was defined as log2(non-silent mutation number + 1). The neoantigen data were downloaded from The Cancer Immunome Atlas (TCIA, https://tcia.at/) (25). The cancer-testis antigen (CTA) scores and homologous recombination deficiency (HRD) scores were downloaded from the supplementary material of a previous publication (24). The intratumor heterogeneity (ITH), defined as the subclonal genome fraction measured by ABSOLUTE, was retrieved from an earlier publication (24). The GISTIC2.0 results of In silico Admixture Removal (ISAR) calibrated SCNV data (minus germline CNV) were obtained from a previous publication (24). The GISTIC2.0 thresholded result of 2 or -2 was defined as deep CN alterations called amplifications and depletions. While the result of 1 or -1 was defined as shallow CN alterations, which were called gains and losses, respectively. The SCNV load was calculated as the percentage of altered cytobands in each sample. T cell & B cell receptor (TCR&BCR) diversity scores (Shannon Entropy, Evenness, and Richness) were obtained from a previous publication (24). The immune cytolytic activity score (CYT), which is highly correlated with CD8+ T cell activation, was defined as the log-average (geometric mean) of GZMA and PRF1 expression as previously described (33).

### 2.8 Mutations and CNVs comparison between subtypes

Mutations and CNVs were compared between every two subtypes. To adjust the difference in mutation burden between subtypes, we adopted the method based on logistic regression as described in a previous publication (23). Specifically, the subtype was modeled as a logistic function as subtype ~ mutation burden + gene mutation status. If the P-value of a specific gene mutation status adjusted for mutation burden less than 0.05, such gene was defined as differentially mutated between subtypes. Notably, gene mutations with an overall mutation rate of less than 2.5% were excluded from the comparison. CNVs were compared similarly. Gains/amplifications and losses/deletions were compared between every two subtypes separately. For gains/amplifications comparison, losses/deletions events were set to zero. The subtype was modeled as a logistic function as subtype ~ CNV burden + gene gain/amplification status. If the P-value of a specific gene gain/amplifications adjusted for CNV burden less than 0.05, such gain/amplification was defined as differentially distributed between subtypes. The losses/deletions comparisons were performed similarly. The above calculations were performed using the “glm” function in R.

### 2.9 Statistical analysis

All statistical analyses were performed in R software, version 3.6.1 (The R Foundation for Statistical Computing, http://www.rproject.org/). Continuous and ordered categorical variables were compared using Student’s t-tests or Kruskal–Wallis tests with *post hoc* pairwise Dunn’s-tests. Unordered categorical variables were compared by Pearson χ^2^ test or Fisher exact test. Spearman correlation analyses were employed to evaluate correlations between continuous variables. Survival analyses were performed using Kaplan–Meier analyses and survival differences between groups were compared by log-rank tests. Prognostic factors were identified by univariate and multivariate Cox regression analyses. Factors with univariate regression P<0.1 were further enrolled in multivariate analyses. A two-sided P<0.05 was considered statistically significant unless additionally stated.

## 3 Results

### 3.1 Microenvironmental cell infiltration pattern in CRC

**Figure 1:**
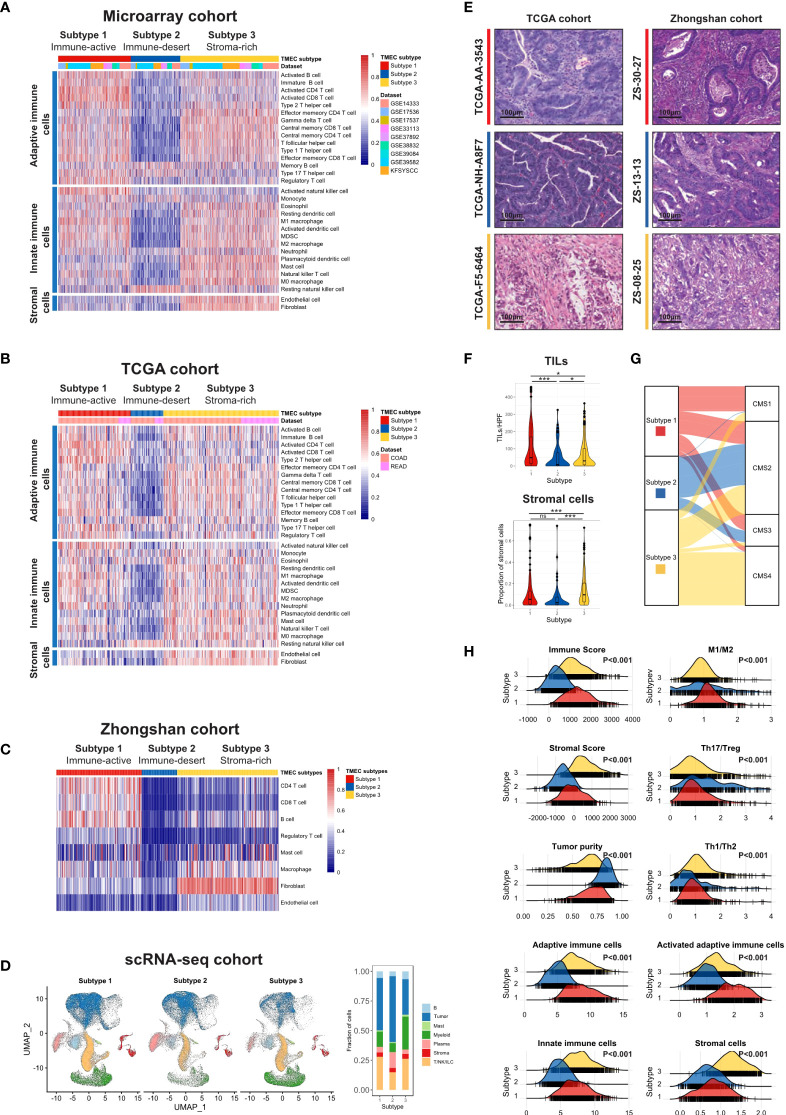
The landscape of TME cell infiltration pattern in CRC. **(A, B)** Heatmaps illustrating the infiltration of 31 TME cells in microarray **(A)** and TCGA **(B)** cohorts. Both were divided into three subtypes by unsupervised clustering. **(C)** Heatmap and clustering of major TME cells infiltration in Zhongshan cohort. **(D)** UMAP of all cells in the scRNA-seq cohort split by TMEC clusters (left) and the fraction of major cell types in three clusters (right). **(E)** The representative pathology presentation of each subtype in the TCGA (18) and Zhongshan cohort (red, subtype 1; blue, subtype 2; yellow, subtype3; from top to bottom). **(F)** The number of TILs (up) and proportion of stromal cells (down) among three subtypes per high power field (HPF, 200x) in the TCGA cohort. **(G)** Sankey plot illustrating the distribution of CMS subtypes and TMEC subtypes in the microarray cohort. **(H)** The distribution of Immune Score, Stromal Score, tumor purity, macrophage M1/M2 ratio, Th17/Treg ratio, Th1/Th2 ratio, adaptive immune cells, activated adaptive immune cells, innate immune cells, and stromal cells among subtypes in the microarray cohort. *P < 0.05; ***P < 0.001; ns, P > 0.05.

We used the ssGSEA algorithm to estimate the absolute infiltration abundance of 31 different TME cells in 1802 samples of the microarray cohort and 619 samples of the TCGA cohort. After normalization, a nonnegative matrix factorization (NMF) algorithm, an unsupervised clustering method, was applied to classify CRC samples into different subtypes based on TME cell infiltration. The optimal cluster number of 3 was selected by the Cophenetic correlation coefficient in both datasets (**Figures S3 and S4**). Thus, we classified 1802 samples into three distinct TME cell (TMEC) subtypes (**Figure 1A**). Subtype 1, named as immune-active subtype (marked by red), was characterized by high infiltration of adaptive and innate immune cells, especially activated adaptive immune cells, such as activated CD4 T cells, activated CD8 T cells, and activated B cells, and low infiltration of stromal cells. Subtype 2, named as immune-desert subtype (marked by blue), featured low infiltration of most immune and stromal cells. And subtype 3, named as stroma-rich subtype (marked by yellow), had high infiltration of adaptive and innate immune cells, as well as stromal cells but low infiltration of activated adaptive immune cells compared with subtype 1. The above infiltration pattern was also validated in the TCGA dataset (**Figure 1B**). To further validate the above immune phenotypes, the major TME composition was evaluated in the Zhongshan cohort by IHC and in the scRNA-seq cohort by dimensional reduction. As the normalized infiltration quantity and main cell types visualized in **Figures 1C**, **D**, and **Figure S6**, respectively, a distinct TME cell infiltration pattern was validated as described above in the Zhongshan and scRNA-seq cohorts.

Moreover, we evaluated the pathological presentation of three distinct subtypes in diagnostic slides of 499 TCGA and 447 Zhongshan samples. The representative pathology features of each subtype were illustrated in **Figure 1E**. As shown in **Figure 1F**, tumor tissue in subtype 1 featured a median stromal proportion with high tumor-infiltrating leukocytes (TILs) infiltration. Subtype 2 had high tumor purity and low TME cell infiltration. And mesenchymal cells infiltrated extensively in subtype 3. Then we evaluated the TILs and stromal cell infiltration pathologically in TCGA diagnostic slides. TILs infiltrated most in subtype 1 and least in subtype 2 (median, 47 vs. 8 vs. 29, P<0.001), while subtype 3 had a significantly higher stromal cell proportion (median, 0.047 vs. 0.037 vs. 0.098, P<0.001).

Furthermore, we explored other characteristics of each subtype. We used the ESTIMATE algorithm to calculate Immune Score, Stromal Score, and tumor purity for each sample. As shown in **Figure 1H**, Subtype 1 had the highest Immune Score, median Stromal Score, and relatively low tumor purity. And subtype 2 featured the lowest Immune Score and Stromal Score but the highest tumor purity. Subtype 3 had a median Immune Score, the highest Stromal Score, and relatively low tumor purity (all P<0.001). Additionally, the macrophage M1/M2 ratio was the highest in subtype 1 (P<0.001), while the Th17/Treg ratio was the highest in subtype 2 (P<0.001). Samples in subtype 3 had the highest Th1/Th2 ratio (P<0.001), and the proportion of activated adaptive immune cells was the highest in subtype 1 (P<0.001). The above characteristics were all validated in the TCGA dataset (**Figure S7**).

### 3.2 TMEC subtypes and clinicopathological features

The correlations between TMEC subtypes and clinicopathological features of the microarray, TCGA, and Zhongshan cohorts were presented in **Tables S4, S5, and S6**, respectively. Subtype 3 had more samples with advanced stages (P<0.001 in the microarray cohort; P=0.001 in the TCGA cohort; P=0.003 in the Zhongshan cohort). Subtype 1 was associated with more right colon cancer (P<0.001 in both microarray and TCGA cohorts; P=0.001 in the Zhongshan cohort). Additionally, the dMMR/MSI-H samples were significantly enriched in subtype 1 (P<0.001 in all cohorts). Moreover, we compared the distribution of Consensus Molecular Subgroups (CMS) (34) and TMEC subtypes, illustrated as Sankey plots in **Figure 1G** and **Figure S8**. Subtype 1 was mainly composed of CMS1 (MSI immune), CMS2 (Canonical), and CMS3 (Metabolic) samples. The majority of subtype 2 samples were from CMS2. CMS2 and CMS4 (Mesenchymal) samples constituted most of subtype 3. The above features were presented in the both microarray and TCGA cohorts.

### 3.3 TMEC subtypes and CRC prognosis

**Figure 2:**
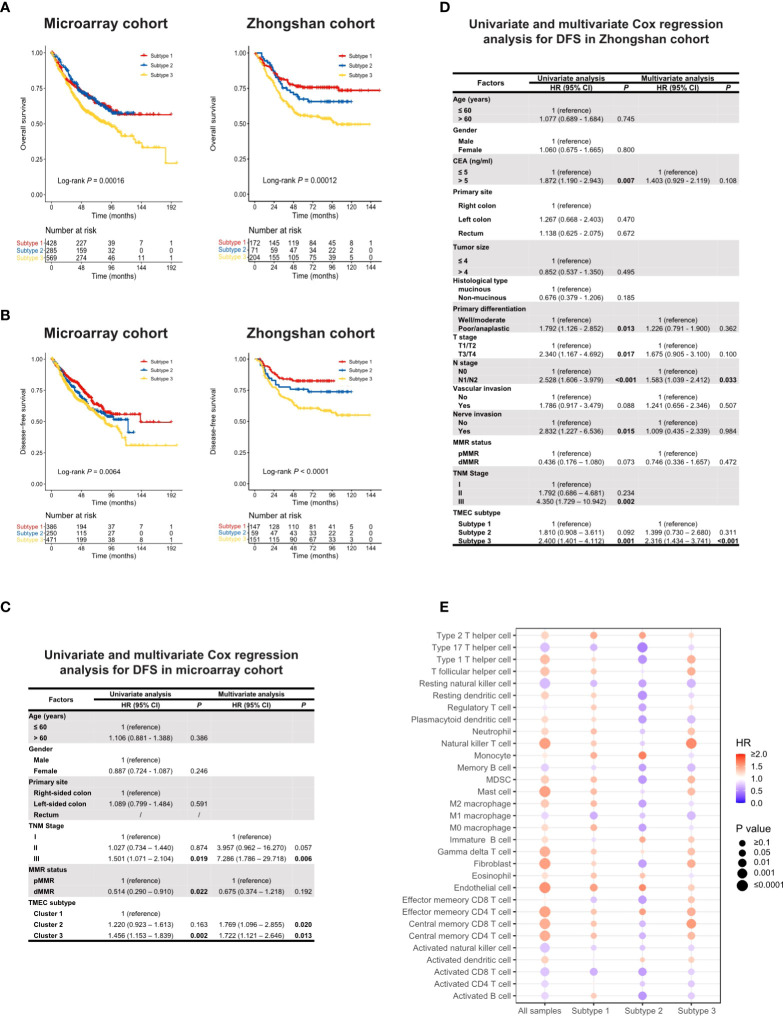
**(A, B)** Kaplan-Meier analysis of OS **(A)** and DFS **(B)** in microarray and Zhongshan cohort. **(C, D)** Univariate and multivariate Cox regression of clinicopathological factors and TMEC subtypes for DFS in microarray **(C)** and Zhongshan cohort **(D)**. **(E)** The prognostic value of different TME cells in all samples and each subtype in the microarray cohort for OS.

TMEC subtypes also play an important role in CRC prognosis. Analyses related to overall survival (OS) were performed in 1281 samples from the microarray cohort and 447 samples from the Zhongshan cohort with available OS data. The analyses related to disease-free survival (DFS) were performed in 1107 samples from the microarray cohort and 357 samples from the Zhongshan cohort of stage I-III with DFS data available. Subtype 3 conferred significantly worse OS and DFS, while subtype 1 and 2 had similar prognoses (**Figures 2A, B**). Next, we conducted univariate and multivariate Cox regression analyses of clinicopathological factors and TMEC subtypes for OS and DFS (**Tables S7, S8**, and **Figures 2C, D**, respectively). Multivariate analyses showed that subtype 3 was an independent prognostic factor of DFS in both the microarray and Zhongshan cohorts (P=0.013, HR=1.722, 95% CI=1.121-2.646; P<0.001, HR=2.316, 95% CI=1.434-3.741, respectively) (**Figures 2C, D**). For OS, subtype 3 only showed independent prognostic value in the Zhongshan cohort (P=0.012, HR=1.636, 95% CI=1.114-2.401) but not in microarray cohort (P=0.195, HR=1.151, 95% CI=0.931-1.423).

Furthermore, we analyzed the prognostic value of different TME cells in all samples and each subtype in the microarray dataset for OS (**Figure 2E**) and DFS (**Figure S9**). Interestingly, the same cell type may play converse prognostic roles in different TMEC subtypes. In subtype 2, the majority of immune and stromal cells were presented as protective factors of survival, even immunoinhibitory cells such as MDSCs and M2 macrophages, which indicated heterogeneous immune networks in different TMEC subtypes.

### 3.4 Possible immune escape mechanism of distinct TMEC subtypes

The distinct characteristics of TMEC subtypes made us wonder whether they had different immune escape mechanisms. Previous studies summarized vital factors leading to tumor immune escape as follows: (1) defective antigen presentation; (2) tolerance and immune deviation; (3) infiltration of immune-suppressive cells; and (4) immune-suppressive mediator secretion (35, 36). As multi-omics data were available for the TCGA dataset, we explored the possible immune escape mechanism of the three TMEC subtypes.

#### 3.4.1 Defective antigen presentation

**Figure 3:**
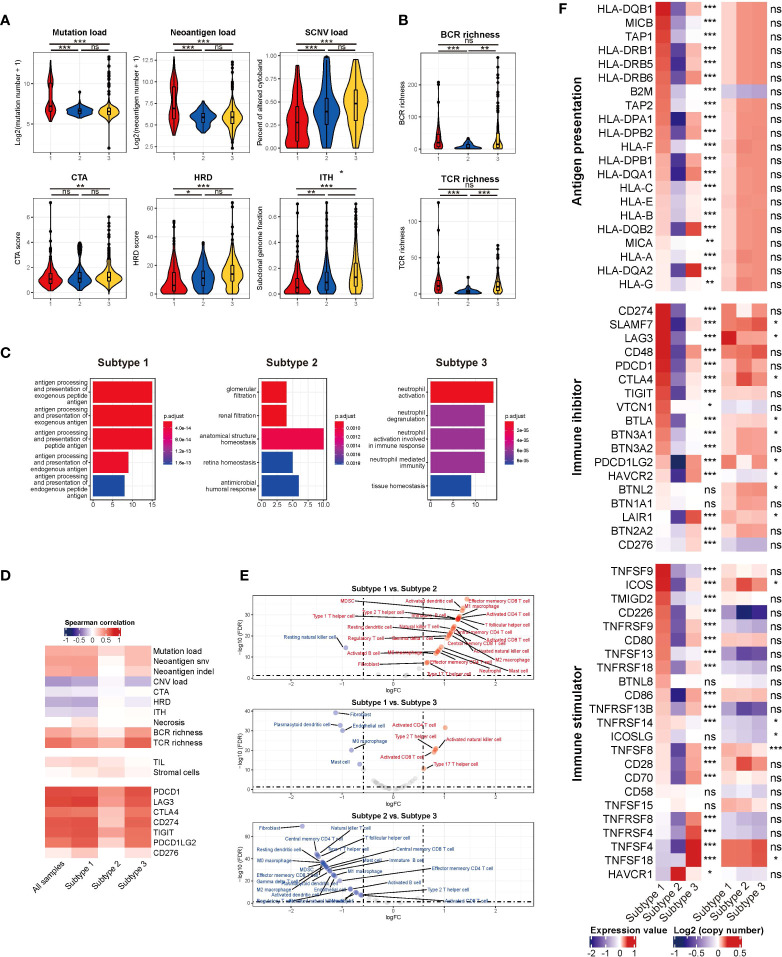
**(A)** The mutation load, neoantigen load, SCNV load, CTA, HRD, and ITH among TMEC subtypes. **(B)** The BCR and TCR richness of different subtypes. **(C)** Pathways enriched in tumor cells among TMEC subtypes in the scRNA-seq cohort. **(D)** The correlation between immune cytolytic activity and tumor immunogenicity factors, TILs, stromal cells, and immune checkpoint molecules. **(E)** The comparison of each cell type between subtypes. **(F)** The relative expression levels to the median value and mean log2(copy number) value of immune inhibitors and immune stimulators among three subtypes. ***P < 0.001; **0.001 < P < 0.01; *0.01 < P < 0.05; ns: P > 0.05.

Defective antigen presentation was composed of at least two aspects: alteration of tumor immunogenicity and down-regulation of antigen presentation pathway. For tumor immunogenicity evaluation, subtype 1 had the highest mutation burden and neoantigen load (**Figure 3A**) (both P < 0.001), which could be easily speculated for the highest proportion of dMMR/MSI-H samples. Subtype 3 featured the highest SCNV load (**Figure 3A**). The CTA and HRD scores of subtype 3 were higher than those of subtype 1 (P = 0.008 and P < 0.001, respectively) but not subtype 2 (both P > 0.05) (**Figure 3A**). Additionally, subtype 3 had the highest ITH level (P < 0.001) (**Figure 3A**). The necrosis level between subtypes was not significantly differentiated (P = 0.167) (**Figure S10**). Subtype 2 showed the lowest BCR and TCR richness diversity (both P < 0.01) (**Figure 3B**), which was positively correlated with cytolytic activity (**Figure 3D**). Pathway enrichment analysis found that antigen presentation pathways were significantly enriched in tumor cells of subtype 1 (**Figure 3C**). For antigen presentation pathway-related gene expression, subtype 1 had higher expression of most MHC-related genes, while the expression of subtype 2 was the lowest (all P < 0.01) (**Figure 3F**). Overall, the three subtypes all had impaired antigen presentation to some extent, but subtype 2 had the lowest immunogenicity and antigen presentation gene expression level.

#### 3.4.2 Tolerance and immune deviation

Tumors were also known to induce immune tolerance by upregulating immune inhibitors and downregulating immune stimulators. The relative expression levels and mean log2(copy number) value of immune inhibitors and immune stimulators were illustrated in **Figure 3F**. Subtype 1 had the highest expression level of most immune inhibitors and some immune stimulators, which indicated pro-tumor and anti-tumor immunity activation. The activation level of most of the above genes was the lowest in subtype 2. Subtype 3 highly expressed TNF/TNF-receptors. Some differentially expressed genes, such as SLAMF7, LAG3, CTLA4, BTLA, TNFSF8, et al. in the two modules might be attributed to SCNV. Correlation analyses found that immune cytolytic activity was positively associated with most immune checkpoint expression (**Figure 3D**). Generally, subtype 1 had a high expression of both immune stimulators and immune inhibitors, whose expression level was the lowest in subtype 2.

#### 3.4.3 Infiltration of immune-suppressive cells

**Figure 4:**
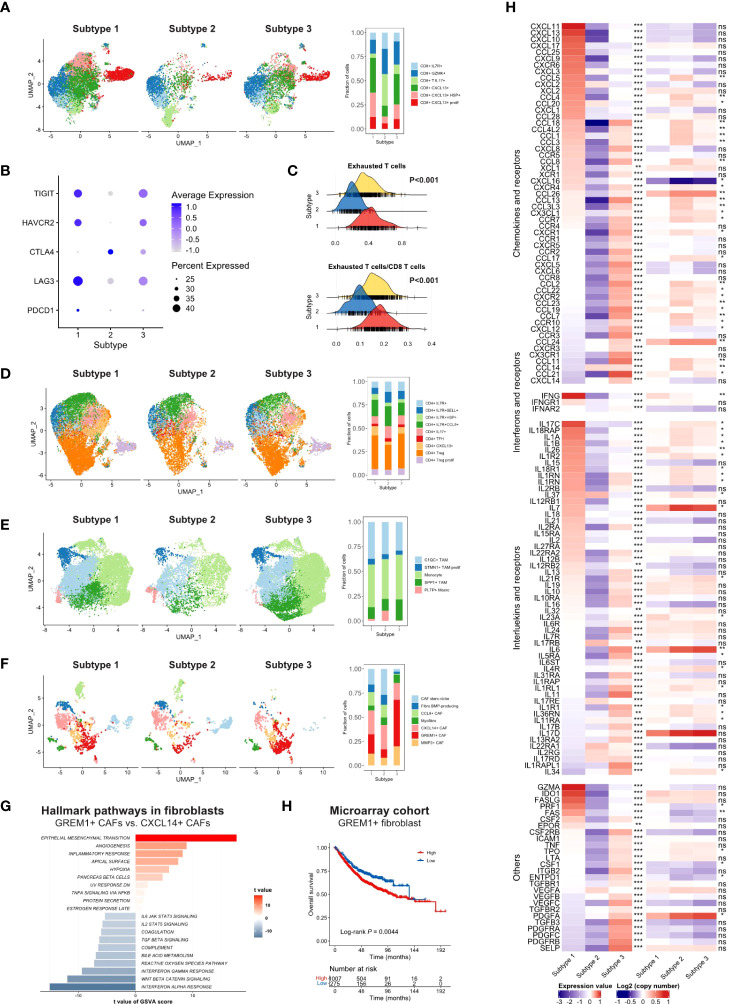
**(A)** UMAP of CD8 T cells in scRNA-seq cohort split by TMEC clusters (left) and the fraction of cell subsets in three clusters (right). **(B)** The average expression of exhaustion markers and expression percentage of CD8 T cells among three clusters. **(C)** The distribution of exhausted T cells infiltration and the ratio of exhausted T cells to CD8 T cells infiltration among each subtype. **(D-F)** UMAP of CD4 T cells **(D)**, monocytes/macrophages **(E)**, and fibroblasts **(F)** in the scRNA-seq cohort split by TMEC clusters (left) and the fraction of cell subsets in three clusters (right). **(G)** GSVA results of hallmark pathways between GREM1+ CAFs and CXCL14+ CAFs. **(H)** Kaplan-Meier analysis of GREM1+ CAFs in microarray cohort. **(E)** The relative expression levels to the median value and mean log2(copy number) value of chemokines, interleukins, other cytokines, and their receptors among three subtypes. ***P < 0.001; **0.001 < P < 0.01; *0.01 < P < 0.05; ns: P > 0.05.

Next, we compared the infiltration of each cell type between subtypes, illustrated as volcano plots (**Figure 3E**). Compared to subtype 2 and subtype 3, subtype 1 had significantly more activated CD4 and CD8 T cell infiltration. Subtype 2, as an immune-cold subtype, had the least adaptive and innate immune cell infiltration among the three subtypes. Subtype 3 had more enriched fibroblasts and endothelial cells than the other two subtypes. Immune-suppressive cells, such as Tregs, MDSCs, and M2 macrophages, were more abundant in subtype 1 and 3. Furthermore, we explored the more detailed difference within major cell types in the scRNA-seq cohort. The proportion of CXCL13+ CD8 T cells was significantly reduced in subtype 2 (**Figure 4A**). The expression level and percentage of exhaustion markers of CD8 T cells were both higher in subtype 1 (**Figure 4B**). Additionally, this such difference was validated at the bulk level. We used the gene signature from a high-quality publication (37) for exhausted T cell infiltration speculation and compared the infiltration of exhausted T cells in each subtype (**Figure 4C**). Subtype 1 had a significantly higher exhausted T cell proportion than the other two subtypes (P < 0.001), as well as a significantly higher ratio of exhausted T cells to CD8 T cells (P < 0.001). The fraction of Tregs was higher in subtype 1 and 3 (**Figure 4D**). For macrophages, subtype 3 had a lower proportion of pro-inflammatory C1QC1+ TAMs and a lower a higher proportion of SPP1+ TAMs (**Figure 4E**), which are involved in angiogenesis (38). A major feature of subtype 3 was extensive infiltration of fibroblasts, scRNA-seq analysis showed that the GREM1+ cancer-associated fibroblasts (CAFs) percentage was significantly higher in this subtype (**Figure 4F**). Further GSVA results showed preferential enrichment of the EMT pathway of GREM1+ CAFs compared to CXCL14+ CAFs (**Figure 4G**). Bulk analysis in the microarray cohort proved that high GREM1+ CAF infiltration was related to a worse prognosis (**Figure 4H**).

#### 3.4.4 Immune suppressive mediator secretion

Furthermore, we investigated the fourth aspect of the immune escape mechanism. The relative expression levels to the median value and mean log2(copy number) value of chemokines and receptors, interferons and receptors, interleukins and receptors, and other cytokines in the three subtypes were compared, and cytokines with P < 0.01 were illustrated in **Figure 4H**. Most cytokine and receptor expression was negatively correlated between subtype 1 and subtype 3, and the expression level of subtype 2 was the lowest on most occasions. Subtype 1 expressed higher levels of pro-immune chemokines, such as CXCL9, CXCL10, and CXCL11, which could recruit effector T cells and NK cells (39). The IFN-γ, which robustly stimulated anti-tumor immunity, was expressed at higher levels in subtype 1. Pro-immunity IL-2 and IL-15 were accumulated in subtype 1. IL-34, CCL2, CCL-3, CXCL8, and some of their receptors CCR5, CXCR1, and CXCR2, which promoted the homeostasis of myeloid cells (40), were expressed extensively in subtype 3. Mesenchymal development-related PDGFs and PDGFRs (41) and cell adhesion-related SELP, ICAM1, and ITGB2 were enriched in subtype 3. However, IDO, which contributed to peripheral tolerance, was significantly highly expressed in subtype 1 (P < 0.001) (**Figure S11A**), indicating the existence of inhibitory cytokines in the inflamed microenvironment. Notably, subtype 2 expressed a significantly higher level of VEGFA. Moreover, STING, a crucial factor in the cGAS–Sting pathway, which contributes to the initiation of innate immunity and recognition of the tumor, was highly expressed in subtype 1 (P < 0.001) (**Figure S11B**), suggesting the impaired immunity initiation in subtype 2 and 3.

### 3.5 TMEC subtype-specific genomic alterations

**Figure 5:**
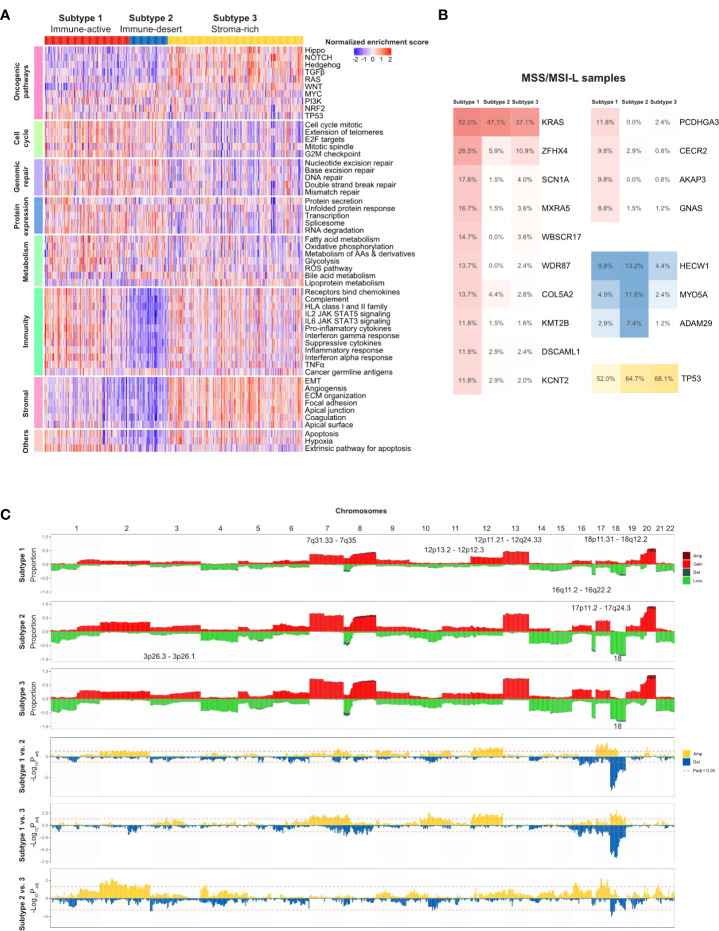
TMEC subtype-specific genomic alterations in the TCGA cohort. **(A)** Heatmap illustrating activation of comprehensive cancer development-related pathways among three TMEC subtypes. **(B)** Subtype-specific gene mutation frequency among MSS/MSI-L samples in three TMEC subtypes. **(C)** Subtype-specific SCNVs of each TMEC subtype (top three plots) and comparison of SCNVs between every two subtypes.

Other than the heterogeneous immune escape mechanism among the TMEC subtypes, we further explored subtype-specific genomic alterations in the TCGA cohort. First, we explored the activation of each subtype in a comprehensive cancer development-related pathway collection (42, 43). As presented in **Figure 5A**, the TP53, NRF2, and PI3K pathways were upregulated in subtype 1, while the MYC and PI3K pathways were enriched in subtype 2. Hippo, NOTCH, Hedgehog, TGF-β, Wnt, and RAS pathways were activated in subtype 3. As for other cancer development-related pathways, cell cycle, genomic repair, protein expression, metabolism, and immunity pathways were generally upregulated in subtype 1. The immunity and stromal pathways, which were highly enriched in subtype 3, were relatively suppressed in subtype 2.

Next, we compared subtype-specific gene mutations in the TCGA cohort. Due to the high percentage of MSI-H patients in subtype 1, it is easy to speculate that most mutations were enriched in subtype 1. However, most mutations in MSI-H patients were random and irregular. Therefore, we focused on the comparison among MSS/MSI-L patients. As shown in **Figure 5B**, after adjusting for mutation burden, KRAS, ZFHX4, SCN1A, MXRA5, WBSCR17, WDR87, COL5A2, KMT2B, DSCAML1, KCNT2, PCDHGA3, CECR2, AKAP3, and GNAS were highly mutated in subtype 1. The mutation frequencies of HECW1, MYO5A, and ADAM29 were higher in subtype 2, while TP53 was mutated more in subtype 3 (all adjusted P <0.05).

As the CNV load of subtype 1 was significantly lower than that of the other two subtypes, the proportion of alterations in most cytobands was the lowest among subtypes (**Figure 5C**). However, after adjusting for CNV burden, most cytobands in Chr12 were more frequently amplified in subtype 1. Additionally, 18p11.31-18q12.2 amplification was enriched in subtype 1. Subtype 2 exhibited more frequent 17p11.2-17q24.3 gain/amplification. Moreover, chr18 loss/deletion was more frequent in subtype 2 and 3.

## 4 Discussion

**Figure 6:**
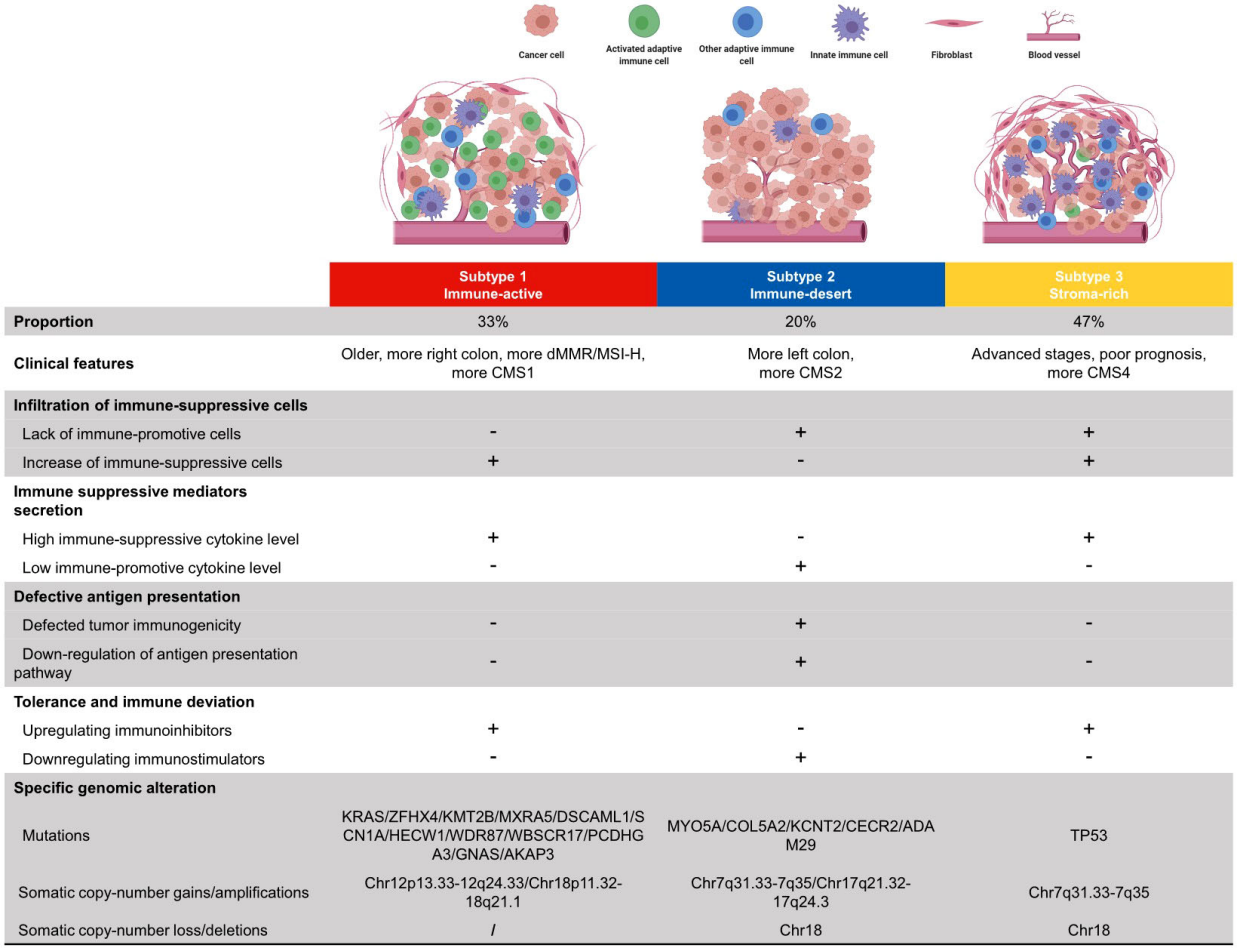
Summary of clinical features, immune escape mechanisms, and subtype-specific genomic alterations of three TMEC subtypes.

The development of personalized cancer medicine has garnered the concern of CRC subtyping. The CRC subtyping system developed from pathological staging and mutation-based subtyping to omics-based subtyping (34). However, the large-scale comprehensive presentation of the CRC TME cell infiltration pattern and subsequent subtyping research were rare and insufficient. This study systematically showed the immune phenotypes of primary TME cells in four independent cohorts for cross-validation. CRC samples can be mainly divided into three subtypes: immune-active, immune-desert, and stroma-rich. The major characteristics of each subtype were summarized in **Figure 6**. The immune-active subtype features highly activated adaptive immune cell infiltrate, more right colon, more dMMR/MSI-H, and more CMS1 samples. The immune-desert subtype was characterized by low infiltration of most TME cells, more left colon, and more CMS2. The stroma-rich subtype presents the distinctiveness of high infiltration of both immune cells and stromal cells, advanced stages, poor prognosis, and more CMS4 samples. Similar TME subtypes can also be observed in ovarian cancer (44) and TNBC (23).

The potential immune escape mechanism of each subtype may have important clinical implications. The immune-active subtype has increased immune-suppressive cells, high immune-suppressive cytokine levels, and upregulated immune inhibitors, implying that both intense tumor-suppressing and tumor-promoting immune responses exist. Theoretically, this subtype may benefit most from immune checkpoint inhibitors (ICIs), the most studied immunotherapy targeting the TME in CRC, and MSI status is a solid predictive marker for ICI efficacy (45). However, not all dMMR/MSI-H patients respond to anti-PD1/anti-PD-L1 therapy. Previous studies reported an ORR of 31% in refractory mCRC (35) and 44% in the first-line setting (46). Most dMMR/MSI-H CRCs are of the immune-active subtype, and targeting the potential immune escape mechanism may be a novel strategy to improve efficacy. Strategies such as erasing the infiltration of immunosuppressive cells, neutralizing immunosuppressive cytokines, and combining other checkpoint inhibitors can be considered to further activate tumor immunity.

The immune-desert subtype has decreased immune-promotive cells, low immune-promotive cytokine levels, downregulation of immune inhibitors, and defective antigen presentation. Nevertheless, OS is similar to the immune-active subtype, implying that the survival impairment of stromal cells has neutralized the survival benefit of high infiltration of activated adaptive immune cells. Even the infiltration of immunosuppressive cells, such as MDSCs and Tregs, may improve prognosis, which shows the complexity and heterogeneity of the CRC TME. The major difference between the immune-desert subtype (“cold tumor”) and the other two subtypes (“hot tumor”) is the antigen presentation defect. Therefore, increasing tumor immunogenicity may induce immune cell chemotaxis and transform a “cold tumor” into a “hot tumor”. Chemotherapy and radiotherapy can cause tumor cell death with the subsequent release of cellular fragmentation and cancer-associated neoantigens, which are presented to APCs to increase tumor immunogenicity (47). The use of pembrolizumab plus FOLFOX achieved an ORR of 55% in the first-line setting (48). However, the synergistic effect of pembrolizumab and radiotherapy was not as expected, which only achieved an ORR of 4.5% in 22 pMMR/MSS mCRC patients (49). Further researches on a dual checkpoint inhibitor (anti-PD-L1 + anti-CTLA-4) following radiotherapy are underway (NCT02701400, NCT03122509). Other novel approaches, such as tumor vaccines, and oncolytic viruses, are under early-phase research.

The stroma-rich subtype, which takes up nearly half of the samples, has a similar immune escape mechanism to the immune-active subtype but decreased immune-promotive cells. This subtype is “hot” but has the worst survival. The primary reason may be the pro-tumoral effect of excessive stromal cell infiltration, excluding activated adaptive immune cells from the tumor. Anti-fibroblast, anti-TGF-β pathway, anti-angiogenesis, anti-immunosuppressive cytokines, or the combination of the above therapy may be expected to transform it into an immune-active subtype. Dual antagonizing of TGF-β and PD-1/PD-L1 showed promising results in preclinical researches (50). The combination of vactosertib (a small-molecule inhibitor of TGF-β) and pembrolizumab showed an ORR of 15.2% in previously treated MSS mCRC patients (51). Furthermore, the REGONIVO (regorafenib + nivolumab) trial reported an inspiring 36% ORR in unselected mCRC patients (52), which implies the potential for ICI and anti-angiogenesis. Previous studies have shown that CAFs secret tumorigenic factors, modulate the immunosuppressive TME, and create an ECM barrier to block CD8 T cells from accessing the tumor (53). Multiple strategies targeting CAFs are under research, such as normalizing the phenotype of CAFs, inhibiting CAF generation and activation, reducing the CAF secretome (54), etc., and awaiting the results.

Additionally, the limitation of this study cannot be ignored. The major one is that the immune escape mechanism speculation was mainly based on in silico analysis, and numerous results only reflected the correlation but not the causation. Further *in vitro* and *in vivo* experiments were needed. Moreover, the unsupervised clustering method limited the clinical application of TMEC subtypes. It is difficult to classify a new sample into a certain subtype. Therefore, we plan to develop a trained classifier and validate it in a large-scale cohort afterwards.

In conclusion, we systematically presented the immune phenotypes of CRC. CRC samples can be divided into three subtypes with distinct TME cell infiltration patterns, clinical features, genomic characteristics, and underlying immune escape mechanisms. The results may provide inspiration and direction for further research on CRC immunotherapy.

## Data availability statement

The raw data supporting the conclusions of this article will be made available by the authors, without undue reservation.

## Ethics statement

The studies involving human participants were reviewed and approved by Clinical Research Ethics Committee of Zhongshan Hospital, Fudan University. The patients/participants provided their written informed consent to participate in this study.

## Author contributions

YM, YX, QF, and JX contributed to the planning of the study. YM, YX, and QF collected the data and performed most of the bioinformatics analysis. YL and WC verified the numerical results by an independent implementation. YM, QF, YX, and YL drafted and revised the manuscript. All authors contributed to the interpretation of data and review of the manuscript. All authors reviewed and approved the final manuscript.

## Funding

This work was supported by the National Natural Science Foundation of China (No. 82002517, 82072678), Shanghai Sailing Program (20YF1407100), Clinical Research Plan of SHDC (SHDC2020CR1033B, SHDC2020CR5006), and Shanghai Science and Technology Committee Project (18140903200).

## Acknowledgments

We would like to thank the staff members of the TCGA Research Network, Gene Expression Omnibus (GEO) repository; as well as all the authors for making their valuable research data public.

## Conflict of interest

The authors declare that the research was conducted in the absence of any commercial or financial relationships that could be construed as a potential conflict of interest.

## Publisher’s note

All claims expressed in this article are solely those of the authors and do not necessarily represent those of their affiliated organizations, or those of the publisher, the editors and the reviewers. Any product that may be evaluated in this article, or claim that may be made by its manufacturer, is not guaranteed or endorsed by the publisher.
